# Mathematical modeling and estimation for next wave of COVID-19 in Poland

**DOI:** 10.1007/s00477-021-02119-5

**Published:** 2021-11-26

**Authors:** M. K. Arti, Antoni Wilinski

**Affiliations:** 1 NSUT East Campus (AIACTR), New Delhi, India; 2 WSB University in Gdansk, Gdansk, Poland

**Keywords:** COVID-19, Prediction, Mathematical Modelling, Gaussian Mixture Model, Pandemics

## Abstract

We investigate the problem of mathematical modeling of new corona virus (COVID-19) in Poland and tries to predict the upcoming wave. A Gaussian mixture model is proposed to characterize the COVID-19 disease and to predict a new / future wave of COVID-19. This prediction is very much needed to prepare for medical setup and continue with the upcoming program. Specifically, data related to the new confirmed cases of COVID-19 per day are considered, and then we attempt to predict the data and statistical activity. A close match between actual data and analytical data by using the Gaussian mixture model shows that it is a suitable model to present new cases of COVID-19. In addition, it is thought that there are N waves of COVID-19 and that information for each future wave is also present in current and previous waves as well. Using this concept, predictions of a future wave can be made.

## Introduction

Currently, the world is threatened by new upcoming of waves of COVID-19, a new disease transmitted by the corona family virus. Many countries around the world have noticed a large number of cases of COVID-19from December 2019 onwards Let us recall some basic facts about the pandemic and recommended behaviors. People with low immunity, aging, and lung-related medical problems are more prone to COVID-19infections (https://www.who.int/emergencies/diseases/novel-coronavirus-2019; Zhou et al. 2020; Wu et al. 2020; Gorbalenya et al. 2020). Symptoms of COVID-19 are coughs, colds, respiratory problems that are very similar to the flu. It is clear from doctors that a person infected with COVID-19 recovers within 14 -16 days because the incubation period of the novel corona virus is 14 days. COVID19 protective measures should be protected by frequent hand washing, to avoid touching the mouth, nose, and face, and to keep the public (2 m or 6 feet) away from other people. COVID-19 is now a pandemic as announced by the World Health Organization (WHO) (https://www.who.int/emergencies/diseases/novel-coronavirus-2019). Therefore, preparation for health care should be adequate worldwide. As the disease is contagious, the number of young infected people is increasing rapidly. If hospitals are not well prepared, health workers will not be able to function properly. In this case, it is inevitable that we will have an accurate measure of new COVID-19 cases, which can help medical managers and administrators. Now three waves of COVID-19have appeared in Poland and in several other countries as well. An important question about the current state of COVID-19, that is, when will the next wave of COVID-19emerge?

The conviction of the Polish authorities is rather optimistic, although very cautious. The daily announcements of the ministry of health (July 2021) predict the fourth wave of the pandemic, most often including fall dates. However, it results rather from premonitions and informal forecasts of an increase in prohibitions, e.g. in connection with the beginning of the school year in the stationary mode planned by the authorities. The anticipated appearance of another wave of infections is also reinforced by the growing aversion to vaccinations of the so far unvaccinated part of the population still below the threshold of general social immunity [in July 2021, about 41% of the population was vaccinated with the second dose (Frank 2020)]. Therefore, virus spread models and prediction methods are still an extremely important challenge for the world of science, including the part that uses statistical inference and artificial intelligence. We consider the data of daily new cases of COVID-19 in this paper and with the help of mathematical modeling, the next wave of COVID-19 is predicted.

In previous publications, the problem of predicting the spread of the virus in Poland has been discussed many times (Wilinski 2021; Bracher et al. 2020; Malesza and Kaczmarek 2021; Bhardwaj and Agrawal 2021), often along with the analysis of the situation in other countries (Mazurek and Neničková 2020; Jaglan et al. 2020; Douglas 2008; Chowdhury et al. 2020). In the above-mentioned studies, statistical data on Poland are directly used to exemplify changes in the spread of COVID-19 (Wilinski 2021; Bracher et al. 2020; Malesza and Kaczmarek 2021; Lalmuanawma et al. 2020; Scheiner et al. 2020). Moreover, there are many studies presenting general models that can be applied to quite any country and the selected data are only a pretext for confirming the methodological correctness of the applied model and the prognostic method (Manav 2020a, 2020b; Koczkodaj 2020; Mazurek and Neničková 2020; Arti 2020; Maziarz and Zach 2020; Wieczorek et al. 2020; Lalmuanawma et al. 2020; Bhardwaj and Agrawal 2021; Scheiner et al. 2020). Some of them are devoted to the analysis of the situation in a selected country, eg in the USA (Sinha and Klahn 2020; Koczkodaj 2020) or India (Arti and Bhatnagar 2020; Roy and Bhattacharya 2020). Interesting and inspiring may be the analysis in terms of the applied models and predictive methods, mainly emerging from artificial intelligence and machine learning algorithms. Reaction–diffusion systems to better comprehend the unlockdown by using SEIR-type model with diffusion to the spatial spread of COVID-19 are proposed in Wilinski and Szwarc (2021); www.pokazwirusa.pl/wykresy; Mammeri 2020).

Data for work were collected from sources such as (https://www.who.int/emergencies/diseases/novel-coronavirus-2019; https://www.worldometers.info/coronavirus/countries; https://ourworldindata.org/COVID-vaccinations; Frank 2020).

In this paper, we propose a Gaussian composite model to present new confirmed cases of COVID-19. Limited estimates can be made using this model. We have read and discussed details of Poland. The proposed model can also be used in other countries as well. Approval intensity is demonstrated by comparing available data with analytical results. In addition, an estimate of the next wave of COVID-19 wave can be made using this model.

## Proposed model

We consider that there are total *N* Gaussian waves. Let us write a mixture Gaussian function with the help of Eq. (1) 

1 $$X\left( t \right) = A_{1} + A_{2} t + \mathop \sum \limits_{k = 1}^{N} p_{k} e^{{ - \frac{{(t - m_{k} ) ^{2} }}{{2sig_{k}^{2} }}}} ,$$

where $A_{1}$ and $A_{2}$ are constants, $e$ is the exponential function; $p_{k}$, $m_{k}$, and $sig_{k}$ are the weight, mean, and standard deviation of k-th Gaussian wave. It can be noticed from (1) that $X\left( t \right)$ contain the information of all *N* Gaussian waves, i.e., all previous, present and future waves. Relation (1) is a general relation and by choosing different values of $p_{k}$, $m_{k}$, and $sig_{k}$, characterization of a particular country can be obtained.

The model given by (1) is showing the nature of pandemic COVID-19, i.e., how it behaves initially, then how it grows and after some time the decay of the disease. It is also telling about the spread and maximum number of infections per day. Further, it shows that initially the rate of infection is linear and slow, e. g., only one COVID-19 patient comes into the country and start spreading the diseases. Since it is infectious disease, it grows exponentially after some time, which is very evident from the model. The parameters in (1), specifically, $p_{k}$, $m_{k}$, and $sig_{k}$ are the maximum number of infections, the day on which maximum number of infections occurred, and spread of k-th COVID-19wave.

$A_{1}$ is the initial number of COVID-19 patients and $A_{2}$ is the rate of change of COVID-19 patients in initial phase.

By looking at the actual data of new active cases of COVID-19 patients in any country, the values of ${A}_{1}$, ${A}_{2}$, N, ${p}_{k}$, ${m}_{k}$, and ${sig}_{k}$ can be obtained by curve fitting. For example, in case of Poland, it can be observed from the actual data of new active cases of COVID-19 that there are three waves of COVID-19, therefore, N = 3 in (1). Further, we have calculated ${A}_{1}$, ${A}_{2}$, N, ${p}_{k}$, ${m}_{k}$, and ${sig}_{k}$ by matching the actual plot of new active cases of COVID-19 patients in Poland with the analytical plot (obtained from (1) with N = 3).

In case of Poland, we can write (1) as

2 $$X_{p} \left( t \right) = 10 + t + 25000 e^{{ - \frac{{(t - 255) ^{2} }}{550} }} + 12000 e^{{ - \frac{{(t - 305) ^{2} }}{350}}} + 32000 e^{{ - \frac{{(t - 390) ^{2} }}{550}}}$$

**Fig. 1:**
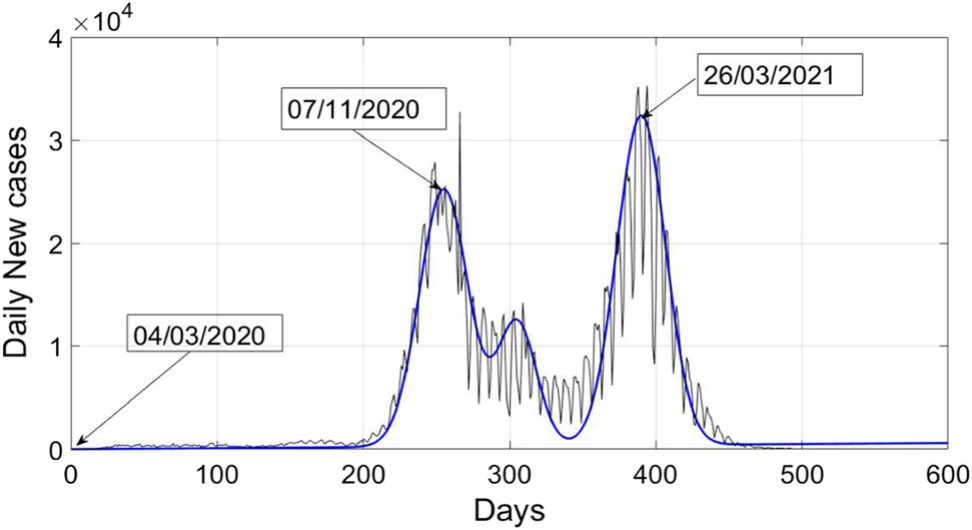
Analytical and actual plot of daily new cases of COVID-19 in Poland

Actual data of daily new cases of COVID-19 in Poland and analytical data is plotted in Fig. 1. Analytical data is calculated by using (2). The actual data of daily confirmed cases in Poland of COVID 19 is considered from 04/03/2020 to 09/07/2021 (https://www.worldometers.info/coronavirus/countries). It can be noticed from actual data (https://www.worldometers.info/coronavirus/countries) that first peak of COVID-19 was observed on 07/11/2020 in Poland with 27,875 fresh cases, whereas the analytical value is approx. 26,570, which is quite close to the true value. Further, a spike can be seen in actual data of value 37,596 on 23/11/2020, it is not modelled properly with the analytical relation given by (2). Then a very small second wave can be observed from Fig. 1, which started in mid of December 2020 and vanished in first week of February 2021. It’s peak value was around 14,216, as seen from the figure. Third wave of COVID-19 started in the month of February 2021, just after second wave. It was more severe as compared to first and second wave. It’s peak value was around 35,246 (https://www.worldometers.info/coronavirus/countries) and it was observed in last week of March 2021. If we calculate the analytical peak of third wave, it is approximately 33,000, which is very close to the actual value.

Therefore, it can be concluded that (2) characterizes the new cases of COVID-19 in Poland efficiently.

We have calculated estimation error between actual data and analytical data by using the following relation:

$$Estimation\;Error = \frac{{Absolute\left( {True\;value - Estimated\;value} \right)}}{{\max \left( {True\;value,\;estimated\;value} \right)}}$$

The estimation error is 0.49 for the considered data of Poland.

## Results of the estimation of the next wave of COVID-19

In this section, it is proposed to estimate the forthcoming COVID-19 wave, in particular, proposing a predictor of the next COVID wave along with its maximum value. To explain this approach, let us first consider the measurement of mean, standard deviation, and the peak value of a Gaussian wave by utilizing its sample values.

### Estimation of mean, standard deviation, and the peak value of a Gaussian wave

**Fig. 2:**
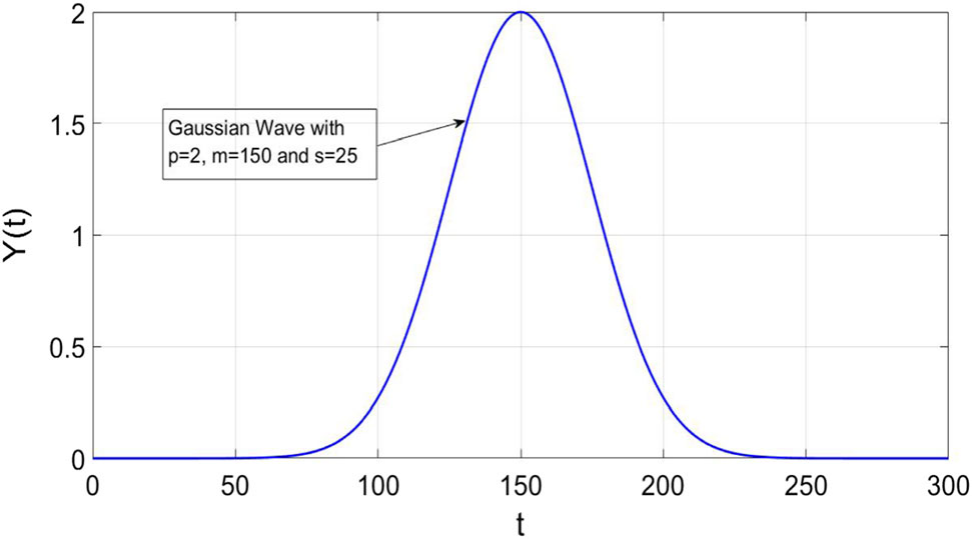
Plot of Gaussian wave

**Fig. 3:**
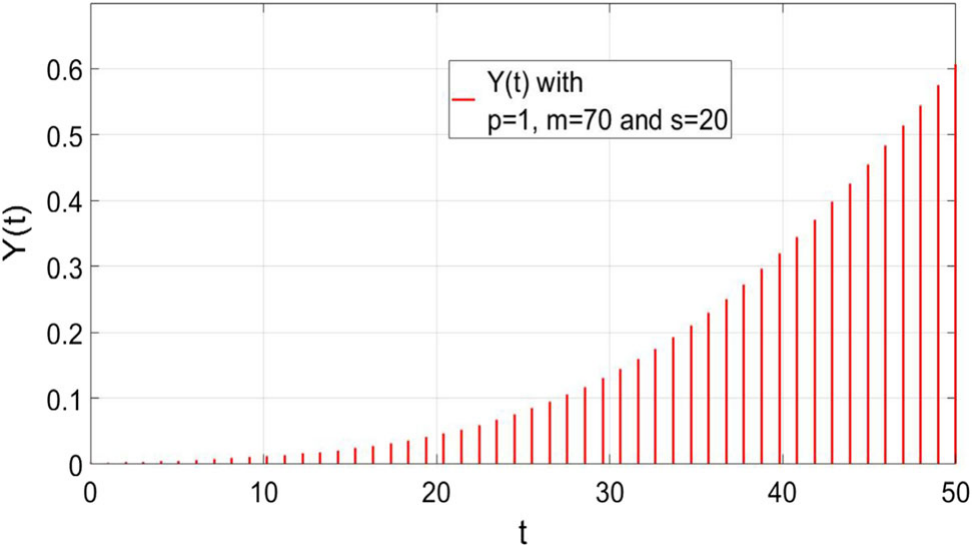
Samples of Gaussian function given by (2)

We can write a Gaussian function as

3 $$Y\left( t \right) = p e^{{ - \frac{{(t - m) ^{2} }}{{2s^{2} }}}} ,$$

where *p, m, s* are the height, mean, and standard deviation of the Gaussian wave provided by (3). The plot of (3) is also shown in Fig. 2. Let us assume that some samples of $Y\left( t \right)$ are available as shown in Fig. 3 and we need to estimate *p, m, s* by using these samples. From (3), we obtain

4 $$Y\left( {t_{1} } \right) = p e^{{ - \frac{{(t_{1} - m) ^{2} }}{{2s^{2} }}}} ,$$

5 $$Y\left( {t_{2} } \right) = p e^{{ - \frac{{(t_{2} - m) ^{2} }}{{2s^{2} }}}} ,$$

and

6 $$Y\left( {t_{3} } \right) = p e^{{ - \frac{{(t_{3} - m) ^{2} }}{{2s^{2} }}}} .$$

By solving (4)–(6) with respect to *p*, *m* and *s* we get

7 $$m = \frac{{\left( {t_{2}^{2} - t_{1 }^{2} } \right)\log_{e} \frac{{Y\left( {t_{2} } \right)}}{{ Y\left( {t_{3} } \right)}} - \left( {t_{3}^{2} - t_{2 }^{2} } \right)\log_{e} \frac{{Y\left( {t_{1} } \right)}}{{Y\left( {t_{2} } \right)}}}}{{2\left( {\left( {t_{2} - t_{1} } \right)\log_{e} \frac{{Y\left( {t_{2} } \right)}}{{Y\left( {t_{3} } \right)}} - \left( {t_{3} - t_{2} } \right)\log_{e } \frac{{Y\left( {t_{1} } \right)}}{{Y\left( {t_{2} } \right)}} } \right)}}$$

8 $$s = \sqrt {\frac{{(t_{1} + t_{2} - 2m)\left( {t_{2} - t_{1} } \right)}}{{2\log_{e} \frac{{Y\left( {t_{1} } \right)}}{{Y\left( {t_{2} } \right)}}}}} .$$

and

9 $$p = \frac{{Y\left( {t_{1} } \right) + Y\left( {t_{2} } \right) + Y\left( {t_{3} } \right)}}{{e^{{ - \frac{{(t_{1} - m) ^{2} }}{{2s^{2} }}}} + e^{{ - \frac{{(t_{2} - m) ^{2} }}{{2s^{2} }}}} + e^{{ - \frac{{(t_{3} - m) ^{2} }}{{2s^{2} }}}} }}.$$

The value of *p* can be obtained by substituting the values of *m* and *s* from (7) and (8), respectively, into (9). Further, it can be observed that only three samples are sufficient to get a Gaussian wave.

### Estimation of mean, standard deviation, and the peak value of a Gaussian wave with N samples

In this subsection, we assume that *N* samples of a Gaussian function is available, as in the case of COVID 19 new wave, in that case following procedure can be used:

1. Divide all samples in the group of three, if it is not possible leave some old samples and take all recent samples, which can make a group of three.
2. Estimate *p, m, s* by using the method given in Sect. 3.1 for all groups*.*
3. Estimate *p, m, s* by performing averaging of *p, m, s* obtained by different groups.

### Prediction of next wave of COVID-19

In the current situation of COVID-19, the most important question is that when will next wave of COVID-19 start for the proper arrangements performed by hospitals and other authorities as well. It can be noticed from (1) that any in any sample of *X(t)* , i.e. from the information of fresh cases of COVID-19, one can have many more details of COVID-19. It is because of the fact that the *X(t)* contains the data of preceding waves, present COVID-19 wave and upcoming COVID-19 waves also. Another important fact is that Gaussian function decreases as it moves away from its mean value, therefore, it can be assumed that the governing waves are only nearby Gaussian waves, all other will contribute negligibly. Let us rewrite (1) in more detailed form as

10 $$X\left( t \right) = A_{1} + A_{2} t + p_{1} e^{{ - \frac{{(t - m_{1} ) ^{2} }}{{2sig_{1}^{2} }}}} + p_{2} e^{{ - \frac{{(t - m_{2} ) ^{2} }}{{2sig_{2}^{2} }}}} + e^{{ - \frac{{(t - m_{3} ) ^{2} }}{{2sig_{3}^{2} }}}} \cdots + p_{N} e^{{ - \frac{{(t - m_{N} ) ^{2} }}{{2sig_{N}^{2} }}}} ,$$

where N can take any positive integer value, large or small. It can be seen from (10) that when the first COVID-19 wave was detected, it contains some information for all future COVID 19 waves. But much of the information is about the second wave of COVID-19, that is, the coming wave. Details of the remaining waves such as third, fourth etc. was very small or overlooked due to the Gaussian nature of the COVID-19 waves. Similarly, when second COVID 19 wave started, these samples contain specific details of the third COVID-19 wave and so on. Using this concept, estimates of the next COVID-19 wave can be made. We begin the measurement process by assuming that some COVID-19 samples are available. The first wave has not yet fully arrived. From (12), we can write

11 $$X_{2} \left( t \right) = A_{1} + A_{2} t + p_{1} e^{{ - \frac{{(t - m_{1} ) ^{2} }}{{2sig_{1}^{2} }}}} ,$$

In (11), $A_{1}$ and $A_{2}$ can be obtained by curve fitting.

By following the method given in III-A, we can find the values of $p_{1} , m_{1}$ and $sig_{1}$. As more samples of first COVID 19 wave received, estimation of $p_{1}$ will be better. Moreover, these values will also supply some details of second COVID-19 wave also. If there is no upcoming COVID-19 wave, then *X(t)* must be zero after the first COVID-19 wave. It shows that after sufficient time, i.e., after dying out the first wave, there must be zero or very small daily new cases of COVID-19 patients. But if it is not so then $X_{2} \left( t \right) - p_{1} e^{{ - \frac{{(t - m_{1} ) ^{2} }}{{2sig_{1}^{2} }}}}$ are the values which may generate second wave of COVID 19. By using these sample values, one can estimate the upcoming COVID-19 wave and so on.

When we have complete information about first wave of COVID-19, it can be used to estimate and validate the second wave. The details of the estimation of second wave are as follows:

After calculating the all parameters of first wave, we can write from (11)

12 $$X_{2} \left( t \right) = 10 + t + 25000 e^{{ - \frac{{(t - 255) ^{2} }}{550} }} .$$

If there is no second/ upcoming COVID-19 wave, then X(t) must be zero after the first COVID 19 wave. It shows that after sufficient time, i.e., after dying out the first wave $\left( {25000 e^{{ - \frac{{(t - 255) ^{2} }}{550} }} \;{\text{will}}\;{\text{tend}}\;{\text{toward}}\;{\text{zero}}} \right)$ there must be zero or very small daily new cases of COVID-19 patients. But if it is not so then.

$X_{2} \left( t \right) - 25000 e^{{ - \frac{{(t - 255) ^{2} }}{550} }}$ are the values which may generate second wave of COVID-19.

It can be observed from Fig. 1 or actual data of COVID-19 patients in Poland that after the peak of first wave, the cases are not tending towards zero. Therefore, there are chances of second wave. Let us consider

13 $$Y_{1} \left( t \right) = X_{2} \left( t \right) - 25000 e^{{ - \frac{{(t - 255) ^{2} }}{550} }} .$$

**Fig. 4:**
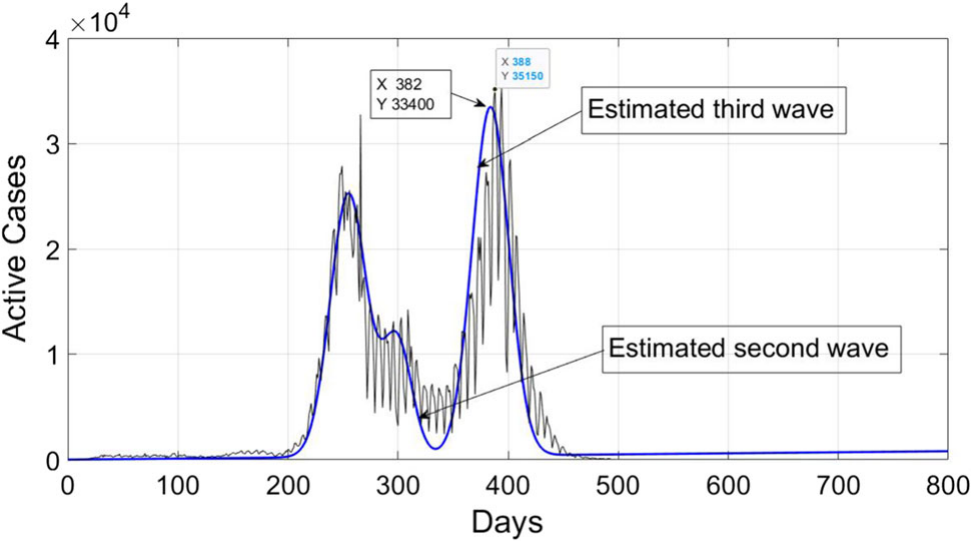
Estimation of second and third COVID 19 wave

Now we consider a set of samples, i.e., actual data from t = 252 to t = 280. Then by using $Y_{1}$(252), $Y_{1}$ (253)…. $Y_{1}$ (280) and following the procedure given in Sect. 3.1, second wave is estimated. The plot of estimated second wave is shown in Fig. 4. It can be seen from the figure that second COVID wave is slightly shifted towards left. It’s estimated mean is 298, whereas actual mean is around 305. Moreover, the peak value of estimated wave is 12170, whereas actual value is around 13,460. Therefore, the number of new active cases with second estimated wave can be written as

14 $$X_{e1} \left( t \right) = 10 + t + 25000 e^{{ - \frac{{(t - 255) ^{2} }}{550}}} + 10156e^{{ - \frac{{(t - 298) ^{2} }}{339}}} .$$

where as the actual mathematical function used till second wave is

15 $$X_{c1} \left( t \right) = 10 + t + 25000 e^{{ - \frac{{(t - 255) ^{2} }}{550}}} + 12000e^{{ - \frac{{(t - 305) ^{2} }}{350}}} .$$

In similar way, we consider the situation during second wave. Let us consider

16 $$Y_{2} \left( t \right) = X_{c1} \left( t \right) - 12000 e^{{ - \frac{{(t - 305) ^{2} }}{350}}} .$$

The sample set from t = 300 to t = 325 is considered. Then by calculating $Y_{2}$ (300), $Y_{2}$(301)…. $Y_{2}$ (325) and following the procedure given in Sect. 3.1, third wave is estimated. The plot of estimated third wave is shown in Fig. 4. It can be observed from the figure that third COVID wave is a approximation of actual one. It’s estimated mean is 382, whereas actual mean is around 388. Moreover, the peak value of estimated wave is 33,400, whereas actual value is around 35,150.

### Estimation of next wave of COVID-19 in Poland

We want to estimate about the next or fourth COVID-19 wave in Poland. From (2), we have the characteristics of the first, second, and third waves of COVID-19. The calculation of the parameters of the second wave and third wave of COVID-19 is performed using the method given in section III-A. The relationship of new COVID-19 cases daily is given in (2).

The main assumptions for estimating the upcoming wave are:

- The social conditions are the same as in third wave of COVID-19, like in terms of social distancing, masking, vaccination, and travel restrictions etc.
- The population live exactly in the same way as during third wave of COVID 19.

If there is no fourth wave then third wave will die out completely and new COVID-19 cases count will be zero. But if it is not so then we need to calculate

17 $$X_{1} \left( t \right) = X_{p} \left( t \right) - 32000 e^{{ - \frac{{(t - 390) ^{2} }}{550}}} .$$

$X_{1} \left( t \right)$ in (12) is the difference between analytical data (calculated by (2)) and data of last COVID-19 wave. Specifically, the third wave in Poland can be written mathematically as $32000 e^{{ - \frac{{(t - 390) ^{2} }}{550}}}$. If we subtract the values generated by this wave (after dying out of third wave) from total number of active COVID-19 patients, we will get the values, which may generate the next wave of COVID-19. Therefore, by using (12), we have a set of values (something like Fig. 3), then by following the method given in Sect. 3.2, the mean, height, and standard deviation can be calculated.

By using the sample values of $X_{1} \left( t \right)$ from 03/04/2021 to 18/05/2021, new COVID wave can be estimated as

18 $$X_{n} \left( t \right) = 10 + t + 25000 e^{{ - \frac{{(t - 255) ^{2} }}{550} }} + 12000 e^{{ - \frac{{(t - 305) ^{2} }}{350}}} + 32000 e^{{ - \frac{{(t - 390) ^{2} }}{550}}} + 15590e^{{ - \frac{{(t - 630) ^{2} }}{350}}} .$$

**Fig. 5:**
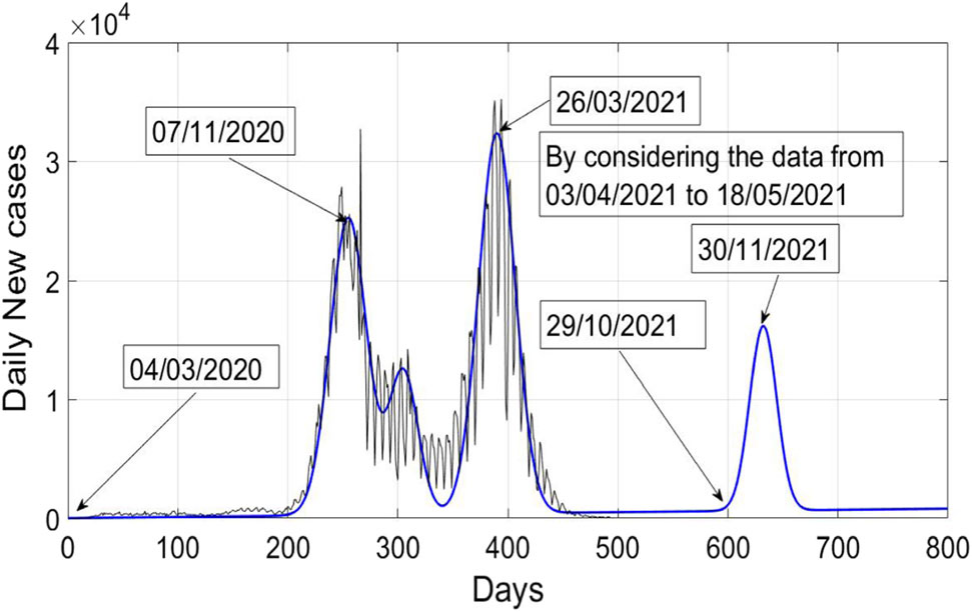
Estimated fourth COVID-19 wave in Poland by considering the new cases from 03/04/2021 to 18/05/2021

Figure 5 shows a plot of analytical and actual cases of daily new cases of COVID-19 in Poland by considering the data of new daily cases from 03/04/2021 to 18/05/2021. Further, it also shows the estimated fourth COVID-19 wave in Poland. It can be observed from Fig. 5 that next or upcoming wave of COVID-19 will be comparatively les severe and it may be expected to appear in the month of October 2021 or November 2021. Moreover, it is very early stage and it is shown by simulations that as well as more samples have been received, a better estimation can be performed. The next COVID-19 wave will also depend upon several other factors like social distancing, use of mask etc. It can be concluded that the estimated value of mean, variance, and height of the Gaussian wave depend largely on the set of samples used.

Similarly, by using the sample values of ${X}_{1}\left(t\right)$ from 03/05/2021 to 07/06/2021, new COVID wave can be estimated as

19 $$X_{n} \left( t \right) = 10 + t + 25000 e^{{ - \frac{{(t - 255) ^{2} }}{550} }} + 12000 e^{{ - \frac{{(t - 305) ^{2} }}{350}}} + 32000 e^{{ - \frac{{(t - 390) ^{2} }}{550}}} + 15330e^{{ - \frac{{(t - 670) ^{2} }}{320}}} .$$

**Fig. 6:**
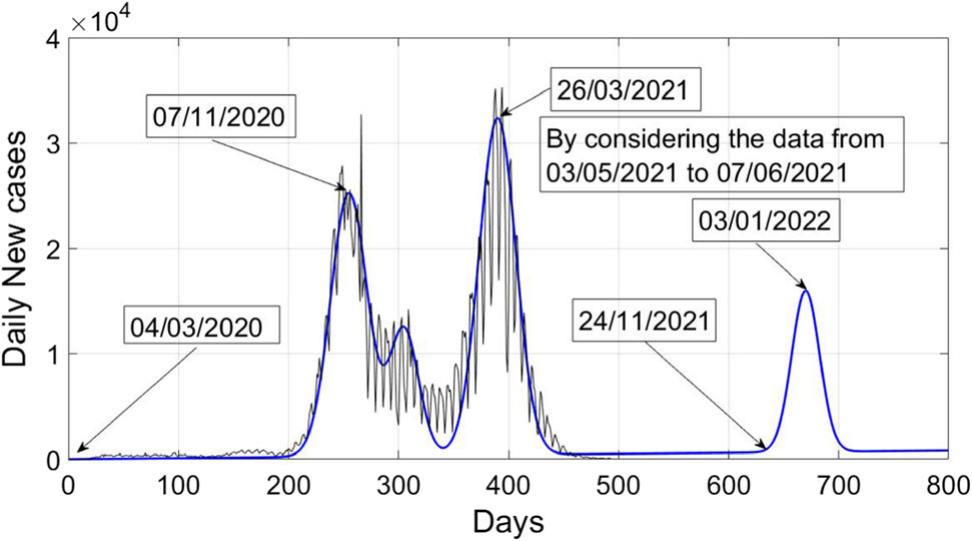
Estimated fourth COVID-19 wave in Poland by considering the new cases from 03/05/2021 to 07/06/2021

Figure 6 shows a plot of daily new cases of COVID-19 versus days in Poland by considering the data of new daily cases from 03/05/2021 to 07/06/2021. Further, it also shows the estimated fourth COVID-19 wave in Poland during the month of November or December 2021. As seen from Figs. 5 and 6, that the prediction of upcoming next wave is largely dependent upon the data samples used. Further, it can also be observed that almost one month delay or advancement can be noticed, if we change set of sample values.

## Discussion and conclusions

A mathematical model has been studied to characterize the COVID-19 daily new cases. A close match between actual and analytical data is evident from numerical results provided in the paper. By choosing different parameter values in the proposed function, the plots can be obtained for other countries also. Specifically, the characterization has been performed of COVID-19 daily new cases in Poland. Moreover, an estimation method has been developed for Gaussian function by utilizing its samples. It can be concluded that the estimated mean, variance and peak of Gaussian function largely depends upon the set of samples used.

The results obtained for Poland do not seem to be in line with the so-called common expectation. However, the authors present and justify the method, obtaining results that are consistent with historical data so far. The model does not take into account many potentially significant predictors that would e.g. occur in regression models (e.g., time series of tests, deaths, recovered, vaccinations, restrictive actions, health service status, etc.). In this matter, it is worth returning to valuable already published and partially cited works (Manav 2020a, 2020b; Roy and Bhattacharya 2020; Koczkodaj 2020; Bhatia and Mitra xxxx; Cherniha and Davydovych 2020; Maziarz and Zach 2020).

Most of the works cited here, those aimed at forecasting methods, limit their aspirations to rather short prediction horizons, often several days, weeks, and less often months, usually stipulating a wide range of the possible forecasted result. However, we show that such far predictive horizons can be achieved, as usual, with the risk of error. We also assume that there are countries for which this method will be more effective than for Poland, as well as those for which it will be less successful. Here is an example of the philosophy of computational intelligence.

With large data set, estimation will also be improved. In addition, the process of estimating the upcoming Gaussian wave has been also proposed. It is shown by estimation that with more samples obtained, a better estimate can be made.
